# Magnetic resonance imaging-guided radiotherapy for portal vein tumor thrombus in hepatocellular carcinoma: outcomes and prognostic factors

**DOI:** 10.1186/s13014-025-02717-5

**Published:** 2025-09-30

**Authors:** So Jung Lee, Myungsoo Kim

**Affiliations:** Department of Radiation Oncology, Incheon St. Mary’s Hospital, College of Medicine, The Catholic University of Korea, Seoul, Republic of Korea

**Keywords:** Portal vein tumor thrombus, Magnetic resonance imaging-guided radiotherapy, Prognostic factors, Hypofractionated radiotherapy, Stereotactic body radiation therapy, Failure pattern

## Abstract

**Background:**

High-dose prescribed radiotherapy has been attempted to improve local control and restore portal vein in patients with hepatocellular carcinoma (HCC) complicated with portal vein tumor thrombus (PVTT). The aim of this study was to evaluate feasibility of real-time tumor-tracking magnetic resonance imaging-guided radiotherapy (rtMRgRT) for PVTT in HCC. In addition, prognostic factors for overall survival (OS) and progression pattern after radiotherapy (RT) were analyzed.

**Methods:**

We retrospectively reviewed the data of 34 patients who had unresectable HCC complicated with PVTT and who were treated with rtMRgRT using hypofractionated radiotherapy (HFRT) and stereotactic body radiation therapy (SBRT) between June 2019 and October 2023. HFRT was performed with a total of 50–60 Gy in 10 fractions, and SBRT was performed in a range of 36–50 Gy in 4–5 fractions. The median biologic effective dose with an a/b ratio of 10 was 100 Gy_10_ (range: 68.4–100 Gy_10_).

**Results:**

Twenty-one patients (61.7%) had an objective response (complete response and partial response) to PVTT; the 1-year estimated local control rate was 77.7%. The median progression-free survival and OS were 5.2 and 10.6 months, respectively. The predominant initial pattern of progressive disease after RT was outfield intrahepatic progression (21/29 cases, 72.4%). RT responder (hazard ratio [HR], 0.33; 95% confidence interval [CI], 0.12–0.88; *p* = 0.026) and combined transarterial chemoembolization (TACE) within 1-month post-RT (HR, 0.24; 95% CI, 0.08–0.73; *p* = 0.012) were favorable prognostic factors for OS.

**Conclusions:**

The rtMRgRT demonstrated feasibility in treatment of PVTT with favorable overall response and local control. Response to RT and combined TACE within a month post-RT were favorable prognostic factors for OS. Given the predominant patterns of disease progression after RT, timely management of HCC outside RT field may be crucial for enhancing the survival of patients with PVTT undergoing RT. The early combination of TACE within a month post-RT may be beneficial in this regard. Further prospective studies are needed to determine the optimal sequencing and timing for combining RT and other local therapies in patients with PVTT.

## Background

Hepatocellular carcinoma (HCC) ranks as the sixth most prevalent cancer [1] and the third leading cause of cancer-related mortality worldwide [2]. Advanced HCC is frequently associated with macrovascular invasion, with portal vein tumor thrombus (PVTT) observed clinically in 35–60% of HCC cases at the time of diagnosis [3–6]. Patients with HCC and PVTT are categorized as having Barcelona Clinic Liver Cancer stage C, with systemic therapy being recommended as the first therapeutic option [7, 8]. However, sorafenib that has been recommended as first line systemic therapy for a long period demonstrated only a modest survival advantage of 3 months for patients with advanced HCC, compared with the placebo group [9, 10]. Recently, several immunotherapeutic agents have been evaluated for patients with HCC [11], and combined immunotherapy has demonstrated improved survival compared to sorafenib in patients with unresectable HCC [12, 13]. In two phase-III randomized controlled trials, the IMbrave150 [12] and HIMALAYA trials [13], the combinations of atezolizumab/bevacizumab and tremelimumab/durvalumab showed significant improvements in overall survival (OS) compared to sorafenib, respectively. These combined immunotherapies are now recommended as first-line systemic therapies for unresectable HCC [7, 14]. However, the HIMALAYA trial excluded patients with main PVTT; therefore, further research is needed regarding the optimal therapeutic option for patients with central PVTT. Radiotherapy (RT) has been shown to improve local control (LC) and induce restoration of portal vein patency in patients with PVTT, which can prevent hepatic deterioration and facilitate subsequent treatments such as transarterial chemoembolization (TACE) or surgical resection [15–17]. Dose-related responses between RT and the treatment outcome in patients with PVTT have been reported in several previous studies [4, 18, 19], and recent studies have explored the effects of stereotactic body radiation therapy (SBRT) for PVTT [16, 20, 21]. Real-time tumor-tracking magnetic resonance imaging-guided RT (rtMRgRT) is a suitable therapeutic option for high-dose prescribed RT, capable of treating tumors accurately while minimizing therapeutic volumes [22, 23]. In 12 patients with HCC, hypofractionated radiotherapy (HFRT) and SBRT using rtMRgRT were previously shown to be effective and safe for PVTT [24]. However, the previous study concentrated on a limited number of patients. The aim of this study was to establish the effectiveness of rtMRgRT for PVTT by analyzing treatment outcomes for a larger number of patients. Additionally, we tried to identify the prognostic factors for OS and analyze the progression pattern post-RT in patients with HCC complicated with PVTT to explore more effective treatment strategies for these patients.

## Methods

### Study design and patient selection

Between June 2019 and October 2023, 50 patients with HCC complicated with PVTT underwent rtMRgRT-HFRT and SBRT using the ViewRay Linac MRIdian system (ViewRay, Cleveland, OH, USA). Of them, 13 patients who were lost to follow-up or with no appropriate follow-up imaging post-RT and three patients with distant metastasis were excluded. Overall, the data of 34 patients were analyzed. The inclusion criteria for this study were as follows: (1) HCC diagnosed histologically or according to imaging criteria of the Korean Liver Cancer Study group [25], (2) Child–Pugh class (CP class) A or B, (3) Eastern Cooperative Oncology Group (ECOG) performance status of 0–2, (4) no history of RT on liver, and (5) RT conducted using a fraction size ≥ 5 Gy. PVTT was evaluated as per the Japanese Vp classification [26]. The CP class, Modified Union for International Cancer Control (mUICC) stages, PVTT classification, lymph node (LN) metastasis, alpha-fetoprotein (AFP) level, biologic effective dose (BED), response of RT, and combined TACE performed within 1-month post-RT completion were investigated for potential OS prognostic factors. Post-RT TACE was implemented in patients without extrahepatic metastasis at the time of evaluation and with an ECOG performance status of 0–1. Regarding liver function, TACE was administered to patients with preserved hepatic function (CP class A or B7), total bilirubin levels < 3 mg/dL, and alanine aminotransferase (ALT) levels < 10 times the upper limit of normal. Patients with uncontrolled ascites or hepatic encephalopathy were considered ineligible for TACE. Continuous variables, such as AFP and BED, were classified into two groups based on values known to be significantly related to the survival of these patients in previous studies [27, 28].

### Radiotherapy

RT was performed in eligible patients with preserved liver function (CP classes A–B) without refractory ascites and with total bilirubin level ≤ 3 mg/dL and aspartate transaminase/ALT levels ≤ 5 times the normal upper limit.

**Fig. 1:**
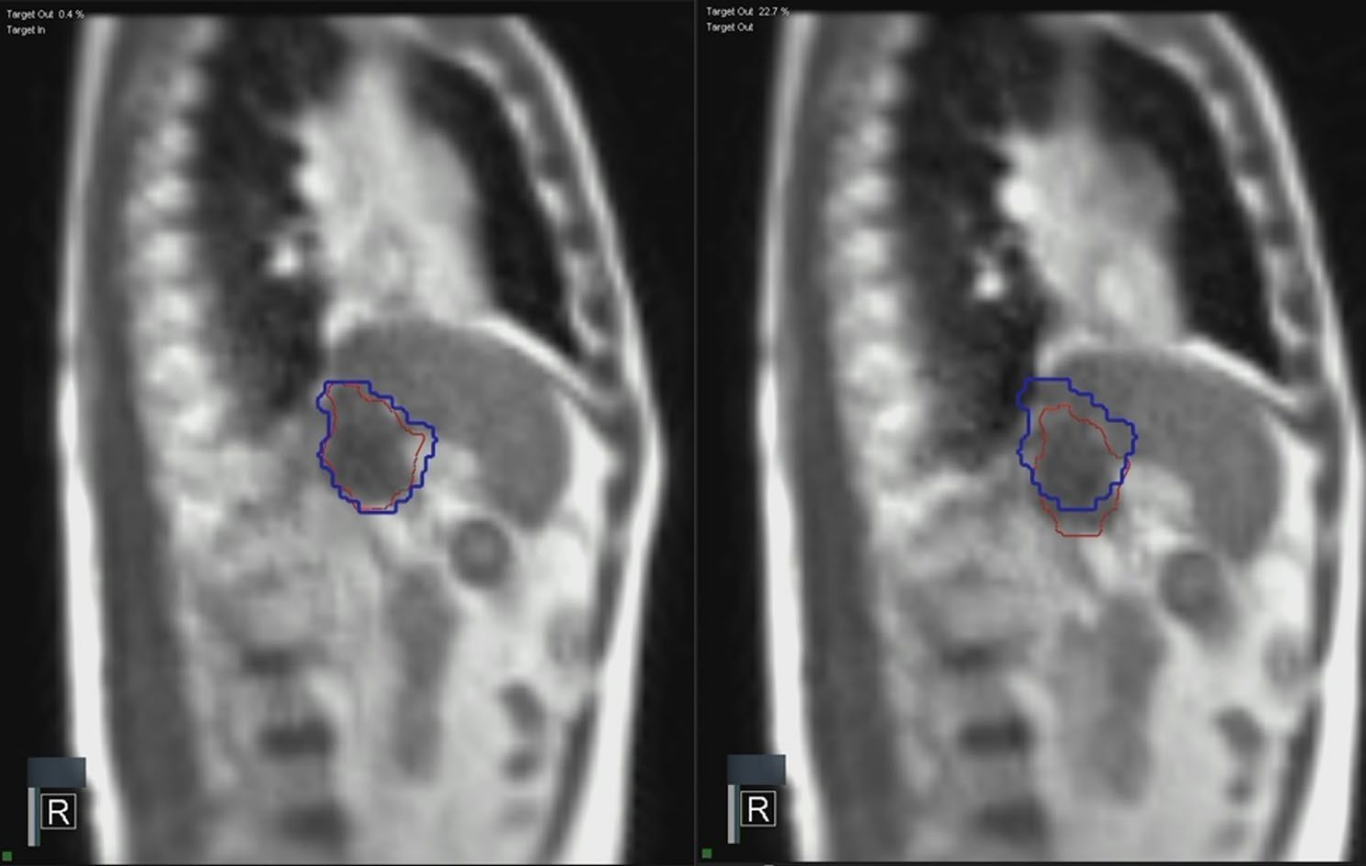
Real time MRI-guided tumor-tracking and gating radiotherapy through CINE MRI. Red line: Target (gross tumor volume of portal vein tumor thrombus), Blue line: Boundary (5 mm margin from target). During radiotherapy, the beam was automatically turned off if the target moved outside the boundary by more than 3%. MRI, magnetic resonance imaging

All patients underwent 0.35-T magnetic resonance imaging (MRI) simulation using the ViewRay Linac MRIdian system (ViewRay, Cleveland, OH, USA) with 3-mm slice thickness. Initial MRI scans were required for 25 s during the end-exhale breath hold phase, and a radiation oncologist confirmed the image quality and target lesion delineation. Post the initial MRI scan, a cine MRI scan was acquired for 10 s using the end-exhale breath hold method. The radiation oncologist then checked the real-time tumor tracking from one selected sagittal plane. Computed tomography (CT) simulation was conducted within 30 min post-MRI simulation completion. Subsequently, the enhanced CT simulation imaging was fused with the MRI scan imaging. Gross tumor volume (GTV) included the PVTT and adjacent HCC mass if it was contiguous. The planning target volume was set at a 5–7-mm margin from the GTV. In RT planning, the prescribed dose was delivered to encompass ≥ 95% of the PTV (D95% = 100%), while respecting normal organ constraints. When the PTV directly abutted the stomach or duodenum, coverage was modified to cover 90% of PTV (D90% = 100%) and maintained at least 95% of GTV (D95%=100%). The maximum dose to the stomach and duodenum was limited to < 35 Gy and < 24 Gy in HFRT and SBRT planning, respectively. For HFRT, the mean dose to normal liver (whole liver-GTV) was planned to be < 23 Gy. In SBRT planning, the mean liver dose was maintained at < 15 Gy, and the volume of normal liver irradiated with < 15 Gy was maintained at ≥ 700 cm^3^. All patients underwent RT through real-time tumor-tracking and gaiting methods for each fraction (Fig. 1). The details of the simulation, planning, and constraints of rtMRgRT were set as described previously [24].

### Follow-up

Post-RT, the first follow-up was performed at a 1-month interval, with subsequent follow-ups occurring at 2–3-month intervals thereafter. Response evaluation was conducted as per the response evaluation criteria in solid tumors version 1.1, utilizing dynamic liver CT or MRI. Complete response (CR) was characterized by the absence of PVTT and target lesions, partial response (PR) was defined as a ≥ 30% reduction in the sum of longest diameter of target lesion of RT, and progressive disease (PD) was indicated by ≥ 20% increase in the sum of longest diameter. Stable disease (SD) is defined as a case of satisfying neither PR nor PD. The median timing of response evaluation was 2.4 months (range, 0.9–4 months) post-RT.

### Statistical analysis

LC was defined as the absence of PD within the RT field (PTV). LC rates and OS were defined as the time from the first day of RT to the occurrence of PD within the RT field and death or last follow-up, respectively. PFS was defined as the duration from the first day of RT to the occurrence of any progression, death, or the last follow-up. LC, OS, and PFS were estimated using the Kaplan–Meier method. Univariate analysis of potential prognostic factors for OS was performed using log-rank test. Multivariate analysis was performed using the Cox proportional hazards model for factors with p-value ≤ 0.05 in univariate analysis. All statistical analyses were performed using R version 4.0.3 (R Development Core Team, Vienna, Austria), and a p-value < 0.05 was considered statistically significant.

## Results

### Patients’ characteristics

The data of 34 patients were analyzed. The median follow-up duration was 8.9 (range, 1.9–28.8) months. The median age of the patients was 63 (range, 42–75) years, with male predominance (91.2%). Most patients had ECOG PS 0–1 (23 patients, 82.4%), and six patients had ECOG 2 status. In total, 28 and six patients had CP class A and B, respectively. The mUICC stage IVA was predominant (24 patients, 70.6%), followed by stage III; two patients had stage II. Large HCC (size > 10 cm) was confirmed in seven patients (20.6%), and multiple HCC mass or infiltrative patterns were observed in 23 patients (67.6%). Thirty patients (88.2%) had concomitant Vp3–4 PVTT, and four patients had concomitant Vp2. The patients’ characteristics are summarized in Table 1.

**Table 1:** Patient characteristics

Factor	No. (%), total 34
Age	Median, 63 years (range, 42–75 years)
Sex	
Male	31 (91.2%)
Female	3 (8.8%)
Etiology	
HBV	23 (67.7%)
HCV	3 (8.8%)
NBNC	8 (23.5%)
ECOG Performance Status	
0–1	28 (82.4%)
2	6 (17.6%)
Child–Pugh classification	
A (5–6)	28 (82.4%)
B (7–9)	6 (17.6%)
Tumor maximum diameter (cm)	Median, 5.8 cm (range, 1.4–18 cm)
< 5 cm	11 (32.3%)
5–10 cm	16 (47.1%)
> 10 cm	7 (20.6%)
Mass number	
Single	11 (32.3%)
Multiple or infiltrative	23 (67.6%)
LN metastasis	
Yes	3 (8.8%)
No	31 (91.2%)
mUICC stage	
II	2 (5.9%)
III	8 (23.5%)
IVA	24 (70.6%)
PVTT classification	
Vp2	4 (11.8%)
Vp3	11 (32.3%)
Vp4	19 (55.9%)
AFP	Median, 216.6 (range, 2.25–151,055.7)
≤ 400 mg/L	20 (58.8%)
> 400 mL/L	14 (41.2%)
PIVKA-II	median, 2163.1 (range, 16.96–30,000)

HBV, hepatitis B virus; HCV, hepatitis C virus; NBNC, non-B non-C hepatitis; ECOG, Eastern Cooperative Oncology Group; LN, lymph node; mUICC, Modified Union for International Cancer Control; PVTT, portal vein tumor thrombus; AFP, alpha-fetoprotein; PIVKA-II, prothrombin-induced by vitamin K absence or antagonist-II

### Treatments

Seven patients received HFRT, and 27 patients received SBRT. HFRT was performed with 50 Gy in 10 fractions for six patients, whereas one patient received 60 Gy in 10 fractions. SBRT was performed between 36 and 50 Gy in 4–5 fractions, and 24 of the 34 patients (70.6%) received 50 Gy in five fractions. The median BED with an a/b ratio of 10 was 100 (range: 68.4–100) Gy_10_; 20 patients were treated with BED ≥ 100 Gy_10_. The median values of GTV and PTV were 28.4 (range: 8.2–903.7) cm^3^ and 77.1 (range: 32.5–1,540.4) cm^3^, respectively. The median liver volume was 1,360.4 (range: 755.1–3,193) cm^3^, and the median mean liver dose was 13.42 (range: 5.83–21.94) Gy. Five patients received RT as the first treatment. Pre-RT, 27 and four patients underwent TACE and radiofrequency ablation, respectively. Five patients had undergone previous systemic therapy. Two patients had no subsequent treatment post-RT. Seventeen patients underwent TACE after RT, among whom 14 underwent combined TACE within a month following RT. Systemic therapy post-RT was performed in 24 patients. The details of the treatments are presented in Table 2.

**Table 2:** Treatments

Previous treatments	No. (%), Total = 34
None	5 (14.7%)
Surgery	4 (11.8%)
TACE	27 (79.4%)
RFA	4 (11.8%)
Sorafenib	2 (5.9%)
Atezolizumab + bevacizumab	3 (8.9%)
**Post-radiotherapy treatments**	
None	2 (5.9%)
TACE	17 (50%)
RFA	1 (25%)
Sorafenib	9 (26.5%)
Regorafenib	2 (5.9%)
Lenvatinib	6 (17.6%)
Atezolizumab + bevacizumab	7 (20.6%)
**Radiotherapy**	
GTV (cm^3^)	Median, 28.4 cm^3^ (range, 8.2–903.7 cm^3^)
PTV (cm^3^)	Median, 77.1 cm^3^ (range, 32.5–1,540.4 cm^3^)
Liver (cm^3^)	Median, 1360.4 cm^3^ (range, 755.1–3,193 cm^3^)
Normal liver (whole liver-GTV, cm^3^)	Median, 1272.4 cm^3^ (range, 717.7–2,017.7 cm^3^)
Target of radiotherapy	
PVTT	25 (73.5%)
PVTT with near HCC mass	9 (26.5%)
HFRT	
50 Gy/10 fractions	6 (17.6%)
60 Gy/10 fractions	1 (2.9%)
SBRT	
36 Gy/4 fractions	1 (2.9%)
40 Gy/5 fractions	2 (5.9%)
50 Gy/5 fractions	24 (70.6%)
BED_10_	Median, 100 Gy_10_ (range, 68.4–100)
Liver Dmean*	Median, 13.42 Gy (range, 5.83–21.94 Gy)
Liver EQD2 Dmean (Gy_3_)	Median, 14.71 Gy_3_ (range, 4.9-22.75 Gy_3_)
Liver V15 **(cm^3^)	Median, 442.73 cm^3^ (range, 67.45–982.01 cm^3^)
Liver V21 ***(cm^3^)	Median, 241.8 cm^3^ (range, 32.79–686.43 cm^3^)

No., number; TACE, transarterial chemoembolization; RFA, radiofrequency ablation; GTV, gross tumor volume; PTV, planning target volume; PVTT, portal vein tumor thrombus; HCC, hepatocellular carcinoma; HFRT, hypofractionated radiotherapy; SBRT, stereotactic body radiation therapy; BED_10_, biologic effective dose of a/b 10, EQD2, equivalent dose in 2 Gy fractions

*Liver Dmean; mean irradiation dose of liver

**Liver V15 (cm^3^); liver volume that was irradiated ≥ 15 Gy

***Liver V21 (cm^3^): liver volume that was irradiated ≥ 21 Gy

### Treatment outcomes and pattern of progression

**Fig. 2:**
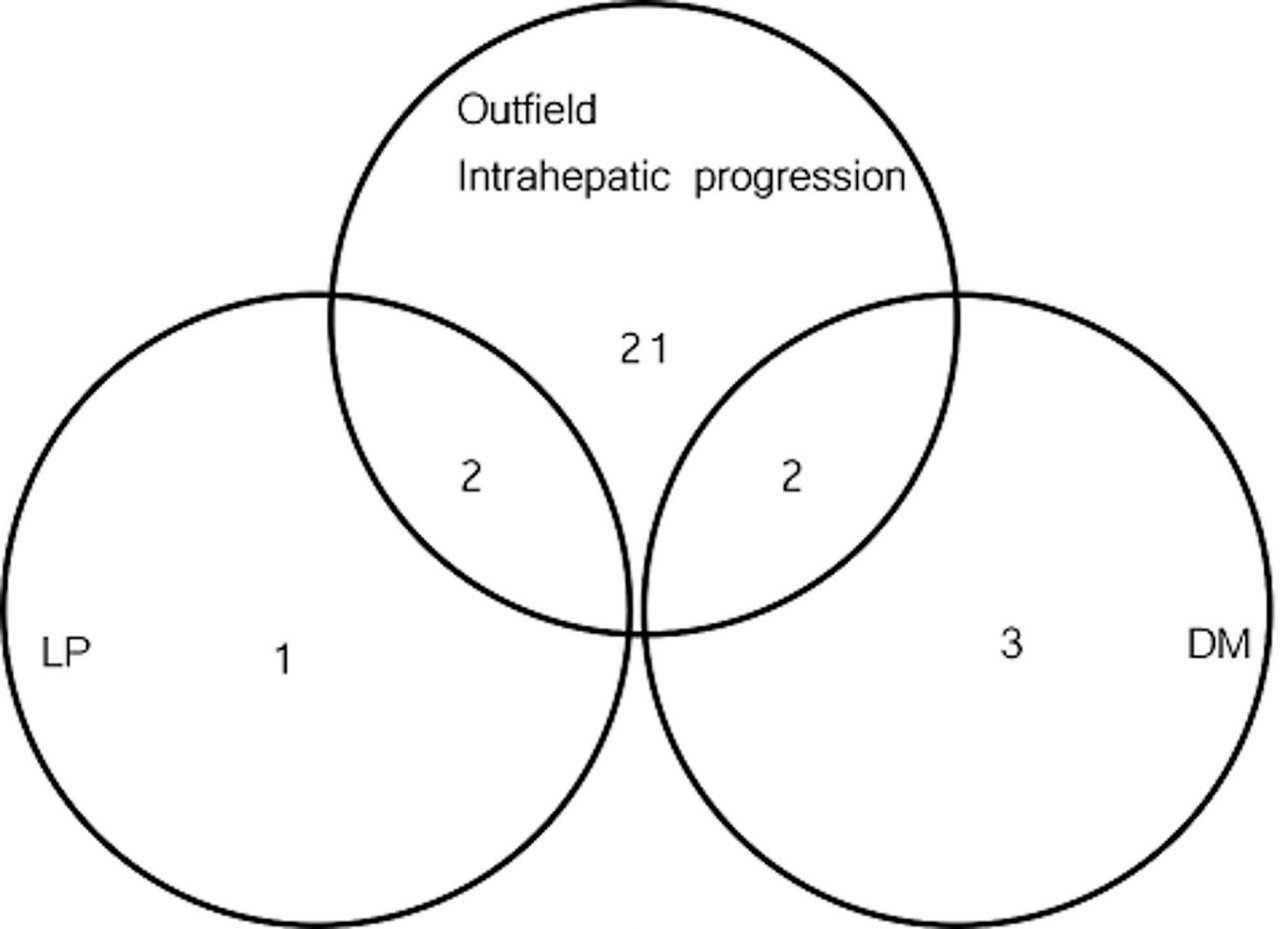
First pattern of progressive disease after radiotherapy. LP, local progression; DM, distant metastasis

In the response evaluation, two and 19 patients showed CR and PR, respectively. The objective response (CR and PR) rate was 61.7%. SD and PD were confirmed in nine (26.5%) and four (11.8%) patients, respectively. The estimated 1-year LC rate was 77.7%. At the time of analysis, 29 patients showed PD, and 28 patients died. The median and 1-year OS were 10.6 (95% confidence interval [CI], 7.53–14.8) months and 41.2%, respectively. The median and 1-year PFS were 5.2 (95% CI, 4.23–9.53) months and 21.1%, respectively. The predominant initial pattern of disease progression post-RT was outfield intrahepatic progression (21/29 cases, 72.4%). Local progression accompanied by outfield intrahepatic progression was observed in two cases. Isolated local progression within RT field, distant metastasis (DM), and outfield intrahepatic progression with DM were confirmed in one, three, and two cases, respectively (Fig. 2).

### Prognostic factors

**Fig. 3:**
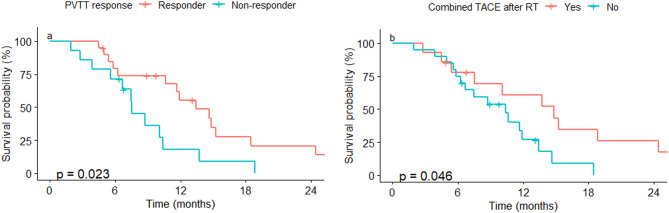
Kaplan−Meier survival curve for overall survival. According to (**a**) response of radiotherapy and (**b**) whether transarterial chemoembolization was combined after radiotherapy PVTT, portal vein tumor thrombus; TACE, transarterial chemoembolization; RT, radiotherapy

In the univariate analysis of OS, patients with CP class B showed a significantly lower 1-year OS than patients with CP class A (22.2% vs. 45%, *p* = 0.03). The higher AFP group (> 400 IU/mL) showed a poorer 1-year OS than the lower AFP group (25.7% vs. 52%, *p* = 0.006). RT responders (CR/PR) showed a significantly higher 1-year OS than non-responders (56.4% vs. 17.6%, *p* = 0.01). TACE, performed within 1-month post-RT, was a favorable OS prognostic factor with marginal significance (1-year OS, 60.6% vs. 26.9%, *p* = 0.05; Fig. 3). The median survival periods of groups with or without combined TACE were 14.8 months and 10.4 months, respectively. The HFRT and SBRT groups had comparable OS rates. The results of our univariate analysis are described in Table 3. In the multivariate analysis, RT responder (hazard ratio [HR], 0.33; 95% CI, 0.12 − 0.88; *p* = 0.026) and follow-up TACE within a month (HR, 0.24; 95% CI, 0.08 − 0.73; *p* = 0.012) post-RT were identified as favorable OS prognostic factors (Table 4).

**Table 3:** Univariate analysis of prognostic factors on overall survival

Factors	Number	6-month OS (%)	1-year OS (%)	*p*-value
**Sex**				0.8
Male	31	73.7	38.2	
Female	3	100	66.7	
**Child–Pugh classification**				0.03*
A	28	82.1	45	
B	6	44.4	22.2	
**mUICC stage**				0.06
II–III	10	100	63.5	
IVA	24	65.8	31.7	
**PVTT classification**				0.7
Vp2	15	73.3	50	
Vp3–4	19	78.6	33.5	
**LN metastasis**				0.2
Yes	3	33.3	0	
No	31	80.4	43	
**AFP**				0.006*
≤ 400 IU/mL	20	100	52	
> 400 IU/mL	14	42.9	25.7	
**Radiotherapy field**				0.5
PVTT only	25	72.0	40.8	
PVTT with near HCC	9	87.5	41.7	
**Radiotherapy technique**				0.09
SBRT	7	71.4	35.5	
HFRT	27	77.4	57.1	
**BED**				0.1
< 100 Gy_10_	10	80	50	
≥ 100 Gy_10_	24	74.6	36.1	
**PVTT response**				0.01*
Responder (CR/PR)	21	80.2	56.4	
Non-responder (SD/PD)	13	69.2	17.6	
**Followed combine TACE**				0.05*
No	20	75	26.9	
Yes	14	77.9	60.6	

OS, overall survival; mUICC, Modified Union for International Cancer Control; PVTT, portal vein tumor thrombus; LN, lymph node; AFP, alpha-fetoprotein; HCC, hepatocellular carcinoma; SBRT, stereotactic body radiation therapy; HFRT, hypofractionated radiotherapy; BED_10_, biologic effective dose; CR, complete response; PR, partial response; SD, stable disease; PD, progressive disease; TACE, transarterial chemoembolization;

*Statistically significant

**Table 4:** Multivariate analysis of prognostic factors on overall survival

Factor		Overall survival	
		HR (95% CI)	*p*-value
CP classification	A/B	2.76 (0.74–10.30)	0.129
AFP (IU/mL)	≤ 400/>400	2.10 (0.87–5.04)	0.095
mUICC stage	II-III/IVA	1.72 (0.64–4.62)	0.28
RT response	Non-responder/responder	0.33 (0.12–0.88)	0.026*
Followed TACE within 1 month	No/Yes	0.24 (0.08–0.73)	0.012*

CP, Child–Pugh; AFP, alpha-fetoprotein; mUICC, Modified Union for International Cancer Control; RT, radiotherapy; TACE, transarterial chemoembolization; HR, hazard ratio; CI, confidence interval

### Toxicity

Three months post-RT, grade 2 liver enzyme elevation was found in six patients. Grade 3 liver enzyme elevation was found in five patients (14.7%). Grade 3 bilirubin elevation was present in five cases (14.7%), wherein three cases occurred with disease progression. No cases of grade 4 toxicity of liver enzyme and bilirubin were observed. A worsened CP score of ≥ 2 within 3 months post-RT was identified in 10 patients; of these, six patients showed elevation by disease progression. Among the four patients (of 34, 11.8%) who experienced a worsened CP score ≥ 2 without disease progression, two recovered to their previous score within 3 months, whereas the other two maintained a CP score of 7. Regarding gastrointestinal toxicity, grades 2 and 1 duodenitis were identified in one case. No cases of treatment-related death or grade-4 toxicity were identified.

## Discussion

This study investigated the response and treatment outcomes of HFRT and SBRT for PVTT through rtMRgRT. In this study, the overall rtMRgRT response and 1-year LC rate for PVTT were 61.7% and 77.7%, respectively. These findings are consistent with those of previous studies analyzing patients with PVTT who received RT, in which overall response ranged from 49.2 to 75.6% [16, 21, 29] and the LC rate ranged from 69.3 to 88.7% [21, 30, 31]. These comparable results suggest the effectiveness of rtMRgRT as RT technique that enables targeting with minimal margin without the need for an invasive strategy such as the fiducial marker insertion.

In this study, RT responders and combined TACE within 1-month post-RT were identified as favorable prognostic factors for OS. The correlation between PVTT response to RT and survival has been reported previously. Shui et al. analyzed the efficacy of SBRT for PVTT and reported distinct survival according to the response of PVTT [16]. Patients exhibiting CR, PR, SD, and PD responses showed a median survival of not reached and 13, 8, and 4 months, respectively. Ji et al. found a significant survival difference between responders and non-responders for PVTT among both groups of patients who were administered SBRT or lenvatinib [32]. Combined TACE within a month post-RT was another favorable OS prognostic factor in this study. The survival benefit of combining RT with TACE has been reported previously [15, 16, 21]. Shui et al. explored the efficacy of SBRT for PVTT and found that the median survivals of the SBRT and TACE combined with SBRT groups were 12.0 ± 1.6 and 3.0 ± 1.0 months, respectively [16]. Choi et al. reported a 1-year OS of SBRT and SBRT with TACE of 14.6% and 71.4%, respectively, and administration of TACE within 3 months post-SBRT as a favorable prognostic factor for survival [21]. In our study, administration of TACE within a month post-RT was identified as a significant timing, improving the OS of patients. The optimal timing and sequencing of combining RT and TACE in patients with HCC has not yet reached a consensus. In a study by Buyn et al. involving patients with HCC who underwent incomplete TACE, the introduction of early RT within 5 weeks was associated with a significant improvement in local failure-free survival compared to delayed RT [33]. In a meta-analysis by Zhang et al. [34] comparing two treatment strategies (SBRT combined with TACE versus TACE alone) in patients with HCC complicated with PVTT, the subgroup with a TACE-SBRT interval ≤ 28 days showed significantly improved OS compared to TACE-only group. In patients with HCC complicated with PVTT, the early combination of SBRT and TACE may serve as an effective strategy to control PVTT while reducing the tumor burden of the HCC mass. The optimal timing and sequencing of combining two modalities should be further elucidated through prospective studies.

The median survival of our cohort was 10.6 months, slightly lower than that reported in previous studies, which range from 10.0 to 20.8 months [16, 21, 29]. Our findings might be attributable to rapid progression of the multiple and advanced HCC mass, as most patients (24/34 patients, 70.6%) were in mUICC stage IVA. In this study, the 1-year LC rate was 77.7%, whereas the 1-year PFS was 21.1%; the most prevalent initial progressive pattern of post-RT was outfield intrahepatic progression (21/29 cases, 72.4%). These survival and progression patterns suggest that in patients with HCC complicated with PVTT, timely treatment of the HCC masses outside the RT field with control of PVTT through high-dose RT may be crucial for survival of patients. Early combination of TACE with RT may be beneficial in this regard.

Regarding toxicity, most patients showed mild radiation side effects and no grade ≥ 3 gastrointestinal toxicity. Grade ≥ 3 toxicities of liver enzymes and bilirubin, without disease progression, were confirmed in 14.7% and 5% of the total cohort, respectively, with no RT-related deaths or liver failure incidents. Additionally, the group of patients who received combined TACE within 1-month post-RT showed tolerable liver toxicity: Grade 3 liver enzyme elevation was confirmed in three cases. Worsening of CP score of ≥ 2 was identified in two patients; of these, one experienced disease progression, whereas the other showed recovery within 3 months. Our findings on the tolerability and safety of SBRT and HFRT for PVTT aligned with previous findings [16, 21, 29].

This study has certain limitations, including a small number of patients as well as the potential selection bias resulting from its retrospective design within a single institution. Some patients (*n* = 13) who underwent rtMRgRT were excluded from the analysis due to loss to follow-up or no appropriate follow-up imaging, which may have introduced selection bias related to missing data. This potential bias could have affected the evaluation of survival and toxicity. In addition, various treatment modalities, such as TACE or systemic therapies other than RT, were performed pre- and post-RT. These factors are inherent limitations of observational studies, and the results of this study should be interpreted with caution.

## Conclusions

The rtMRgRT demonstrated feasibility in treatment of PVTT with favorable overall response and LC. RT responders and combined TACE within a month following RT are identified as favorable prognostic factors for OS in rtMRgRT for PVTT. Given the predominant patterns of disease progression post-RT, timely management of HCC outside RT field may be crucial for enhancing the survival of patients with PVTT undergoing RT. The early combination of TACE within a month post-RT may be beneficial in this regard. Further prospective studies are needed to determine the optimal sequencing and timing for combining RT and other local therapies.

## Data Availability

No datasets were generated or analysed during the current study.

## Abbreviations

OS: overall survival
rtMRgRT: real-time tumor-tracking magnetic resonance imaging-guided radiotherapy
PVTT: portal vein tumor thrombus
HCC: hepatocellular carcinoma
HFRT: hypofractionated radiotherapy
SBRT: stereotactic body radiation therapy
TACE: transarterial chemoembolization
CR: complete response
PR: partial response
PD: progressive disease
SD: stable disease
ECOG: Eastern Cooperative Oncology Group
GTV: gross tumor volume
MRI: magnetic resonance imaging
RT: radiotherapy
CP class: Child–Pugh classification
mUICC: Modified Union for International Cancer Control
LN: lymph node
AFP: alpha-fetoprotein
BED: biologic effective dose
ALT: alanine aminotransferase
CT: computed tomography
LC: local control

## References

[1] Bray F, Laversanne M, Sung H, Ferlay J, Siegel RL, Soerjomataram I, et al. Global cancer statistics 2022: GLOBOCAN estimates of incidence and mortality worldwide for 36 cancers in 185 countries. CA Cancer J Clin. 2024;74:229–63. 38572751 10.3322/caac.21834

[2] Rumgay H, Arnold M, Ferlay J, Lesi O, Cabasag CJ, Vignat J, et al. Global burden of primary liver cancer in 2020 and predictions to 2040. J Hepatol. 2022;77:1598–606. 36208844 10.1016/j.jhep.2022.08.021 PMC9670241

[3] Li L-Q, Zhou Y, Huang Y, Liang P, Liang S-X, Su T-S. Stereotactic body radiotherapy versus intensity-modulated radiotherapy for hepatocellular carcinoma with portal vein tumor thrombosis. Hep Intl. 2021;15:630–41. 10.1007/s12072-021-10173-y 33818714

[4] Sun JX, Shi J, Li N, Guo WX, Wu MC, Lau WY, et al. Portal vein tumor thrombus is a bottleneck in the treatment of hepatocellular carcinoma. Cancer Biol Med. 2016;13:452–8. 28154776 10.20892/j.issn.2095-3941.2016.0059 PMC5250602

[5] Cerrito L, Annicchiarico BE, Iezzi R, Gasbarrini A, Pompili M, Ponziani FR. Treatment of hepatocellular carcinoma in patients with portal vein tumor thrombosis: beyond the known frontiers. World J Gastroenterol. 2019;25:4360–82. 31496618 10.3748/wjg.v25.i31.4360 PMC6710186

[6] Cheng S, Chen M, Cai J, Sun J, Guo R, Bi X, et al. Chinese expert consensus on multidisciplinary diagnosis and treatment of hepatocellular carcinoma with portal vein tumor thrombus (2018 Edition). Liver Cancer. 2020;9:28–40. 32071907 10.1159/000503685 PMC7024893

[7] Ducreux M, Abou-Alfa GK, Bekaii-Saab T, Berlin J, Cervantes A, de Baere T et al. The management of hepatocellular carcinoma. Current expert opinion and recommendations derived from the 24th esmo/world Congress on Gastrointestinal cancer, barcelona, 2022. ESMO Open. 2023;8. 10.1016/j.esmoop.2023.101567 PMC10245111 37263081

[8] 2022 KLCA-NCC Korea practice guidelines for the management of hepatocellular carcinoma. J Liver Cancer. 2023;23:1–120. 37384024 10.17998/jlc.2022.11.07 PMC10202234

[9] Llovet JM, Ricci S, Mazzaferro V, Hilgard P, Gane E, Blanc JF, et al. Sorafenib in advanced hepatocellular carcinoma. N Engl J Med. 2008;359:378–90. 18650514 10.1056/NEJMoa0708857

[10] Cheng AL, Kang YK, Chen Z, Tsao CJ, Qin S, Kim JS, et al. Efficacy and safety of Sorafenib in patients in the Asia-Pacific region with advanced hepatocellular carcinoma: a phase III randomised, double-blind, placebo-controlled trial. Lancet Oncol. 2009;10:25–34. 19095497 10.1016/S1470-2045(08)70285-7

[11] Mandlik DS, Mandlik SK, Choudhary HB. Immunotherapy for hepatocellular carcinoma: current status and future perspectives. World J Gastroenterol. 2023;29:1054–75. 36844141 10.3748/wjg.v29.i6.1054 PMC9950866

[12] Finn RS, Qin S, Ikeda M, Galle PR, Ducreux M, Kim TY, et al. Atezolizumab plus bevacizumab in unresectable hepatocellular carcinoma. N Engl J Med. 2020;382:1894–905. 32402160 10.1056/NEJMoa1915745

[13] Abou-Alfa GK, Lau G, Kudo M, Chan SL, Kelley RK, Furuse J, et al. Tremelimumab plus durvalumab in unresectable hepatocellular carcinoma. NEJM Evid. 2022;1:EVIDoa2100070. 38319892 10.1056/EVIDoa2100070

[14] 2022 KLCA-NCC Korea practice guidelines for the management of hepatocellular carcinoma. Clin Mol Hepatol. 2022;28:583–705. 36263666 10.3350/cmh.2022.0294 PMC9597235

[15] Yoon SM, Ryoo BY, Lee SJ, Kim JH, Shin JH, An JH, et al. Efficacy and safety of transarterial chemoembolization plus external beam radiotherapy vs Sorafenib in hepatocellular carcinoma with macroscopic vascular invasion: A randomized clinical trial. JAMA Oncol. 2018;4:661–9. 29543938 10.1001/jamaoncol.2017.5847 PMC5885246

[16] Shui Y, Yu W, Ren X, Guo Y, Xu J, Ma T, et al. Stereotactic body radiotherapy based treatment for hepatocellular carcinoma with extensive portal vein tumor thrombosis. Radiat Oncol. 2018;13:188. 30253783 10.1186/s13014-018-1136-5 PMC6157064

[17] Pandey S, Pareek V, Kumar R, Gupta A, Kunhiparambath H, Shalimar, et al. Role of stereotactic body radiation therapy in portal vein tumor thrombosis in hepatocellular carcinoma: A prospective single Institute experience. Int J Radiation Oncology*Biology*Physics. 2023;117:e330–1.

[18] Iwamoto H, Nomiyama M, Niizeki T, Shimose S, Shirono T, Nakano M, et al. Dose and location of irradiation determine survival for patients with hepatocellular carcinoma with macrovascular invasion in external beam radiation therapy. Oncology. 2019;96:192–9. 30650415 10.1159/000495568

[19] Kim N, Cheng J, Huang WY, Kimura T, Zeng ZC, Lee VHF, et al. Dose-Response relationship in stereotactic body radiation therapy for hepatocellular carcinoma: A pooled analysis of an Asian liver radiation therapy group study. Int J Radiat Oncol Biol Phys. 2021;109:464–73. 33229165 10.1016/j.ijrobp.2020.09.038

[20] Kumar R, Yadav HP, Thaper D, Kamal R, Gupta A, Kirti S. Efficacy and toxicity of SBRT in advanced hepatocellular carcinoma with portal vein tumor thrombosis - a retrospective study. Rep Pract Oncol Radiother. 2021;26:573–81. 34434573 10.5603/RPOR.a2021.0048 PMC8382078

[21] Choi HS, Kang KM, Jeong BK, Jeong H, Lee YH, Ha IB, et al. Effectiveness of stereotactic body radiotherapy for portal vein tumor thrombosis in patients with hepatocellular carcinoma and underlying chronic liver disease. Asia Pac J Clin Oncol. 2021;17:209–15. 32757461 10.1111/ajco.13361

[22] Ladbury C, Amini A, Schwer A, Liu A, Williams T, Lee P. Clinical applications of magnetic Resonance-Guided radiotherapy: A narrative review. Cancers (Basel). 2023;15. 10.3390/cancers15112916 PMC10251893 37296879

[23] Witt JS, Rosenberg SA, Bassetti MF. MRI-guided adaptive radiotherapy for liver tumours: visualising the future. Lancet Oncol. 2020;21:e74–82. 32007208 10.1016/S1470-2045(20)30034-6

[24] Lee SJ, Kim M, Kwak YK, Kang HJ. MRI-guided radiotherapy for PVTT in HCC patients: evaluation of the efficacy and safety. J Cancer Res Clin Oncol. 2022;148:2405–14. 34490584 10.1007/s00432-021-03788-z PMC11801031

[25] 2018 Korean Liver Cancer Association-National Cancer Center Korea Practice Guidelines for the Management of Hepatocellular Carcinoma. Korean J Radiol. 2019;20:1042 – 113. 10.3348/kjr.2019.0140 PMC6609431 31270974

[26] Ikai I, Yamamoto Y, Yamamoto N, Terajima H, Hatano E, Shimahara Y, et al. Results of hepatic resection for hepatocellular carcinoma invading major portal and/or hepatic veins. Surg Oncol Clin N Am. 2003;12:65–75. ix. 12735130 10.1016/s1055-3207(02)00082-0

[27] Sun J, Zhang T, Wang J, Li W, Zhang A, He W, et al. Biologically effective dose (BED) of stereotactic body radiation therapy (SBRT) was an important factor of therapeutic efficacy in patients with hepatocellular carcinoma (≤ 5 cm). BMC Cancer. 2019;19:846. 31455251 10.1186/s12885-019-6063-9 PMC6712687

[28] Ma W-j, Wang H-y. Teng L-s. Correlation analysis of preoperative serum alpha-fetoprotein (AFP) level and prognosis of hepatocellular carcinoma (HCC) after hepatectomy. World J Surg Oncol. 2013;11:212. 23981851 10.1186/1477-7819-11-212 PMC3844510

[29] Lee SM, Choi JH, Chie EK, Kang HC, Kim KS. Efficacy and safety of Image-Guided hypofractionated radiotherapy for hepatocellular carcinoma with portal vein tumor thrombosis. Int J Radiation Oncology*Biology*Physics. 2023;117:e313–4. 10.1186/s12885-025-13739-3 PMC12010600 40254568

[30] Hu Y, Zhou M, Tang J, Li S, Liu H, Hu J, et al. Efficacy and safety of stereotactic body radiotherapy combined with camrelizumab and apatinib in patients with hepatocellular carcinoma with portal vein tumor thrombus. Clin Cancer Res. 2023;29:4088–97. 37556120 10.1158/1078-0432.CCR-22-2592

[31] Lee SM, Choi JH, Yoon J-H, Kim YJ, Yu SJ, Lee J-H, et al. Efficacy and safety of image-guided hypofractionated radiotherapy for hepatocellular carcinoma with portal vein tumor thrombosis: a retrospective, multicenter study. BMC Cancer. 2025;25:736. 40254568 10.1186/s12885-025-13739-3 PMC12010600

[32] ji X, Zhang A, Duan X, Wang Q. Stereotactic body radiotherapy versus lenvatinib for hepatocellular carcinoma with portal vein tumor thrombosis: a propensity matching score analysis. Radiat Oncol. 2024;19:143. 39394613 10.1186/s13014-024-02527-1 PMC11468427

[33] Byun HK, Kim N, Seong J. Optimal timing of radiotherapy after incomplete transarterial chemoembolization for Barcelona clinic liver cancer stage B hepatocellular carcinoma. Yonsei Med J. 2021;62:409–16. 33908211 10.3349/ymj.2021.62.5.409 PMC8084693

[34] Zhang X-f, Lai L, Zhou H, Mo Y-j, Lu X-q, Liu M, et al. Stereotactic body radiotherapy plus transcatheter arterial chemoembolization for inoperable hepatocellular carcinoma patients with portal vein tumour thrombus: A meta-analysis. PLoS ONE. 2022;17:e0268779. 35594278 10.1371/journal.pone.0268779 PMC9122181

